# The effect of Temperature-Humidity Index (THI) on the number of stillbirths and stress-to-comfort ratio of sows at different parities in a tropical environment

**DOI:** 10.1007/s00484-026-03240-2

**Published:** 2026-10-06

**Authors:** Amanda Aparecida Brito, Natascha Almeida Marques da Silva, Edmilson Rodrigues Pinto, Ana Luísa Neves Alvarenga Dias, Mara Regina Bueno de Mattos Nascimento

**Affiliations:** 1 Faculty of Veterinary Medicine and Zootechnics, Federal University of Uberlândia, Uberlândia, Minas Gerais Brazil; 2 Institute of Mathematics and Statistics, Federal University of Uberlândia, Uberlândia, Minas Gerais Brazil

**Keywords:** Pig, Heat stress, Thermal comfort, Reproductive performance, Thermoneutral zone, Piglet

## Abstract

It is well known that heat stress negatively affects the productivity, reproduction, and welfare of pigs. However, the role of thermal environments and stress-to-comfort (S/C) ratios in predicting stillbirths has not been studied in tropical regions. Therefore, this study aimed to model and estimate the impact of the maximum Temperature-Humidity Index (THImax) on the number of stillborn piglets and to calculate the S/C ratio. A total of 36,880 farrowings from sows on a commercial farm in Brazil were analyzed. The females were grouped by parity into gilts, primiparous, and multiparous with 2–5 and ≥ 6 farrowings. On the day of farrowing, THImax was classified as follows: ≤74 (thermal comfort), 75–78 (mild stress), 79–82 (moderate stress), and > 82 (severe stress). The effect of THImax on the number of stillbirths per day was estimated using quasi-Poisson regression. The models were adjusted using the quasi-Akaike information criterion (QAIC) and Pearson’s residual variance to select the most appropriate model for the data. Also to analyze the mean number of stillbirths per farrowing by THImax category using one-way ANOVA. Then, the S/C ratio was calculated by THImax category. Of the 1,826 total days, 14.95% were classified as comfortable. According to the model, multiparous sows (≥ 2 farrowings) had more stillbirths per day under THImax > 82, while in primiparous sows, ITUmax < 74 is associated with the highest number of stillbirths per day, with a reduction as ITUmax approaches 74. With the severe S/C ratio, an increase in stillbirths per litter was observed in gilts (20%), primiparous (7%), 2–5 parities (14%), and ≥ 6 parities (19%). The quasi-Poisson model indicated an increase in daily stillbirths in multiparous sows (≥ 2 farrowings) under THImax > 82. The severe S/C ratio was greater than 1 in all parity groups, indicating an increase in the average number of stillbirths per farrowing, mainly under THImax > 82.

## Introduction

According to the Intergovernmental Panel on Climate Change (IPCC 2023), climate change will become increasingly evident, with an increase in the frequency of heat waves and periods of drought, primarily in tropical regions. Projections indicate an increase in moderate to extreme heat stress in Brazil, especially during spring and summer. This intensifies concerns about the impact of climate change on the country’s animal production (Ferreira et al. 2024). Climate change is therefore expected to directly impact pig farming. Adult pigs exhibit behavioral, physiological, and metabolic changes when subjected to high temperatures in an attempt to minimize the effects of heat stress (Shephard and Maloney 2023). However, these adaptations negatively impact the productive and reproductive performance of pigs (Mayorga et al. 2020).

Additionally, the genetic selection of sows for greater prolificacy has been linked to an increased susceptibility to high temperatures, as well as a higher incidence of stillbirths (Udomchanya et al. 2019; Brito et al. 2022). Several factors can influence the number of stillbirths, including high ambient temperature near farrowing, which can increase the number of stillborn piglets (Wegner et al. 2016; Tani et al. 2016). In temperate environments, sows that farrow during the hottest months are at a higher risk of stillbirth due to heat stress (Rangstrup-Christensen et al. 2016; Tani et al. 2016; Zhao et al. 2022).

Although studies have analyzed the impact of heat stress on stillbirths, most were conducted in temperate regions, such as Belgium (Vanderhaeghe et al. 2010), Germany (Wegner et al. 2016), and Japan (Tani et al. 2016). In these regions, an increase in temperature above 22 °C at the time of birth has been reported to increase the risk of stillbirth (Vanderhaeghe et al. 2010), as have increases in temperature and the maximum Temperature-Humidity Index (THImax) in the five days prior to farrowing (Wegner et al. 2016). Tani et al. (2016) developed statistical models in a temperate environment to examine and quantify the effect of temperature and humidity on the number of stillbirths during hot, humid periods and cold periods. The researchers also found that maximum temperature in summer and minimum temperature in winter were associated with an increase in stillbirths.

In temperate regions, heat stress in livestock is assessed by calculating the summer-to-winter ratio (S/W), or the difference in production performance between summer and winter (Guinn et al. 2019). In tropical regions, however, the seasons are not well defined, and temperature varies within the same month. Thus, this ratio strategy can be used to evaluate performance on days considered to be heat stress (stress, S) divided by performance on days considered to be thermal comfort (comfort, C) (Brettas et al. 2025).

It is essential to understand the impact of the thermal environment on the risk of stillbirths through statistical modeling using THImax in order to guide producers in making management decisions during hot periods, develop effective strategies to mitigate the harmful effects of heat stress, and improve reproductive indices and animal welfare, particularly in tropical regions. This study aimed to model the association between THImax on the day of farrowing and the occurrence of stillbirths in sows in a tropical environment. Additionally, we estimated the effect of THImax on the daily number of stillbirths in each parity group using the model’s generated equations. In addition, the mean number of stillborn piglets per farrowing was calculated for each parity group under different THImax categories, as well as the Stress/Comfort ratio for mild, moderate, and severe stress conditions relative to the comfort condition (C). These findings may help producers make better decisions about sow management during hot periods in intertropical regions, particularly as climate change leads to hotter conditions.

## Materials and methods

A commercial piglet farm provided reproductive data from 38,452 farrowings that occurred from January 2019 to December 2023. The study was conducted on a single commercial swine farm, and all sows were housed in a single farrowing facility. However, only data from natural farrowings (i.e., those that were not induced) were retained for analysis, resulting in 36,880 observations. The farm is located in the Triângulo Mineiro mesoregion in the state of Minas Gerais, Brazil. According to the Köppen climate classification, the region has a tropical climate with a dry winter (Aw) (Reboita et al. 2015). The region is characterized by five dry months (May to September) and a rainy season (November to March). June is the coldest month with an average temperature ranging from 18 to 19 °C, and October is the hottest month with an average temperature of 24.2 °C (Novais et al. 2018; Petrucci 2018).

The herd consisted of F1 Landrace and Large White sows. The gestation sheds had individual crates equipped with automatic feeders and nipple drinkers. Fans and misters were manually activated during the hottest hours of the day. The farm uses artificial insemination. Previously, Brito et al. (2022) described more information on the gestation pens and sow insemination management.

Five days before farrowing, the sows were transferred to the farrow room and kept in individual farrowing pens. The facility had automated feeders and nipple drinkers for the sows, as well as feed pushers and feeders for the piglets’ solid feed. Curtains in each maternity room improved the housing conditions for the females according to the temperature outside the shed.

The information provided included sow identification, parity group, date of farrowing, number of stillborn piglets, and total farrow size. On the farm, piglets that were born dead but appeared normal were considered stillborn, assuming they died close to, during, or shortly after birth. The number of stillbirths per day was considered the total number of stillborn piglets recorded in all farrowings that occurred on the same day. Based on parity, the sows were divided into four groups, according to Brito et al. (2022): gilts (never farrowed), primiparous, 2–5 farrowings, and ≥ 6 farrowings.

According to Carrara et al. (2023), meteorological information was obtained from the National Aeronautics and Space Administration/Prediction of Worldwide Energy Resources (NASA/POWER) platform (http://power.larc.nasa.gov) and included hourly air temperature and relative humidity data from 2019 to 2023, considering the geographical coordinates of the farm. The highest temperature (Tmax, °C) and lowest relative humidity (RHmin, %) were selected for each day to calculate the maximum daily Temperature-Humidity Index (THImax) (Ekine-Dzivenu et al. 2020) by be the most critical variables to quantify heat stress (Ravagnolo et al. 2000). The THImax was calculated using the equation proposed by Berman et al. (2016):

 THImax=3.43+1.058*Tmax-0.293*RHmin+0.0164*Tmax*RHmin+35.7

Where:

Tmax is the highest air temperature obtained from 24 hourly measurements;

RHmin is the lowest relative humidity obtained from 24 hourly measurements.

The daily THImax was classified into four categories adapted from Mellado et al. (2018): ≤74 (thermal comfort, TC), 75–78 (mild stress, MS), 79–82 (moderate stress, MoS), and > 82 (severe stress, SS). For each farrowing analyzed, the THImax category on the day of farrowing was considered.

A descriptive analysis was performed to characterize reproductive performance and the thermal environment. The stillbirth rate was calculated to verify the occurrence of stillbirths in each parity group: (number of stillbirths/number of births) x 100. The generalized linear model (GLM) was used for statistical modeling because it allows for the modeling of response variables with distributions that are not necessarily normal, such as Poisson, binomial, negative binomial, or gamma. This approach is employed when data do not satisfy the assumptions of classical linear regression, particularly when dealing with counts, proportions, or asymmetric variables (see McCullagh and Nelder 1989; for example). We tested Poisson, binomial, negative binomial, and quasi-Poisson regression models beforehand to determine the best fit for the association between THImax and the occurrence of stillbirths. Model fits were evaluated using the quasi-Akaike information criterion (QAIC) and Pearson’s residual variation to ensure the selection of the most appropriate model for the data. The quasi-Poisson regression model was ultimately deemed the best fit for count data with overdispersion. An independent model was adjusted for each parity group and THImax category to allow for a specific assessment of the impact of heat stress on each group. Independence was confirmed by the residuals versus index plot and by the Durbin–Watson test. The quasi-Poisson regression model structure for the frequency of stillbirths as a function of the independent variable (THImax) was defined as follows:

$$\:log\left(\lambda\:\right)=\beta\:0+\beta\:1\left(THImax\right)$$

Where:

λ: mean number of stillbirths;

β0: intercept;

β1: coefficient of the independent variable (THImax).

The number of stillbirths was estimated only for groups in which the quasi-Poisson regression model was statistically significant (*p* < 0.05). For these groups, the predicted number of stillbirths was calculated by exponentiating the model’s predicted values according to the following equation:

$$\widehat Y=e^{\left(\beta\:0+\beta\:1\left(THImax\right)\right)}$$

This approach allowed us to more accurately quantify the effect of THImax on the occurrence of stillbirths.

We performed an analysis of variance (ANOVA) to evaluate the effect of heat stress on the mean number of stillbirths per farrow within each THImax category (thermal comfort, mild stress, moderate stress, and severe stress) and for each parity group (gilt, primiparous, 2–5 farrowings, and ≥ 6 farrowings). Regarding the assumptions of ANOVA, independence is justified by the fact that, within the same THImax classification, the death of one piglet does not affect the others, and normality is asymptotically achieved due to the large number of observations within each THImax category. The assumption of homogeneity of variances across THImax categories was not met; therefore, Welch’s one-way test was applied. When significant differences were identified (*p* < 0.05), the means were compared using Games Howel test.

Additionally, the stress-to-comfort ratio (S/C) was calculated based on the mean number of stillbirths per parity group and THImax class. For each parity group, the average number of stillbirths under mild stress was divided by the average number of stillbirths under comfort (MS/C). The same was done for moderate and severe stress. This method was adapted from Brettas et al. (2025). A S/C ratio of 1.0 indicated no statistical difference between the stress and comfort groups. All analyses were performed using R software (version 4.3.1) with a 5% significance level.

## Results

The percentage of stillbirths per farrow was 7.25%, 5.73%, 7.38%, and 10.41% for gilts, primiparous sows, sows with 2–5 farrowings, and sows with ≥ 6 farrowings, respectively. Primiparous sows had the lowest average number of stillbirths per farrowing. The highest average number of stillbirths per farrowing occurred in sows with ≥ 6 farrowings. Females with 2–5 farrowings had the highest number of piglets per farrowing, and females with ≥ 6 farrowings had the lowest (Table 1).

**Table 1:** Descriptive statistics of the number of stillborn piglets and total piglets per farrowing for gilts, primiparous sows, sows with 2–5 farrowings, and sows with ≥ 6 farrowings, from 2019 to 2023, on a commercial farm in a tropical environment

Parity group	Variable	*N*. of piglets	*N*. of farrowings	Mean ± SE	Minimum	Maximum
Gilts	Stillborns/farrowing	7007	6964	1.01 ± 0.02	0	17
	Total piglets/farrowing	96,637	6964	13.88 ± 0.04	1	29
Primiparous	Stillborns/farrowing	4786	5989	0.80 ± 0.02	0	18
	Total piglets/farrowing	83,491	5989	13.94 ± 0.05	1	27
2–5	Stillborns/farrowing	17,734	16,561	1.07 ± 0.01	0	21
	Total piglets/farrowing	240,070	16,561	14.50 ± 0.03	1	27
≥ 6	Stillborns/farrowing	10,460	7366	1.42 ± 0.02	0	19
	Total piglets/farrowing	100,456	7366	13.64 ± 0.05	1	29

The average number of farrowings per day was 4.19 for gilts, 3.78 for primiparous sows, 9.49 for sows with 2–5 farrowings, and 4.55 for sows with ≥ 6 farrowings. Primiparous sows had the lowest average number of stillbirths per day, while sows with 2–5 farrowings had the highest (Table 2).

**Table 2:** Descriptive statistics of the number of stillbirths per day for gilts, primiparous sows, sows with 2–5 farrowings, and sows with ≥ 6 farrowings, from 2019 to 2023, on a commercial farm in a tropical environment

Parity group	N*	Mean ± SE	Minimum	Maximum
Gilts	1659	4.22 ± 0.11	0	43
Primiparous	1582	3.03 ± 0.08	0	24
2–5	1745	10.16 ± 0.20	0	56
≥ 6	1618	6.47 ± 0.16	0	44

* N is the number of days on which at least one farrowing occurred

**Fig. 1:**
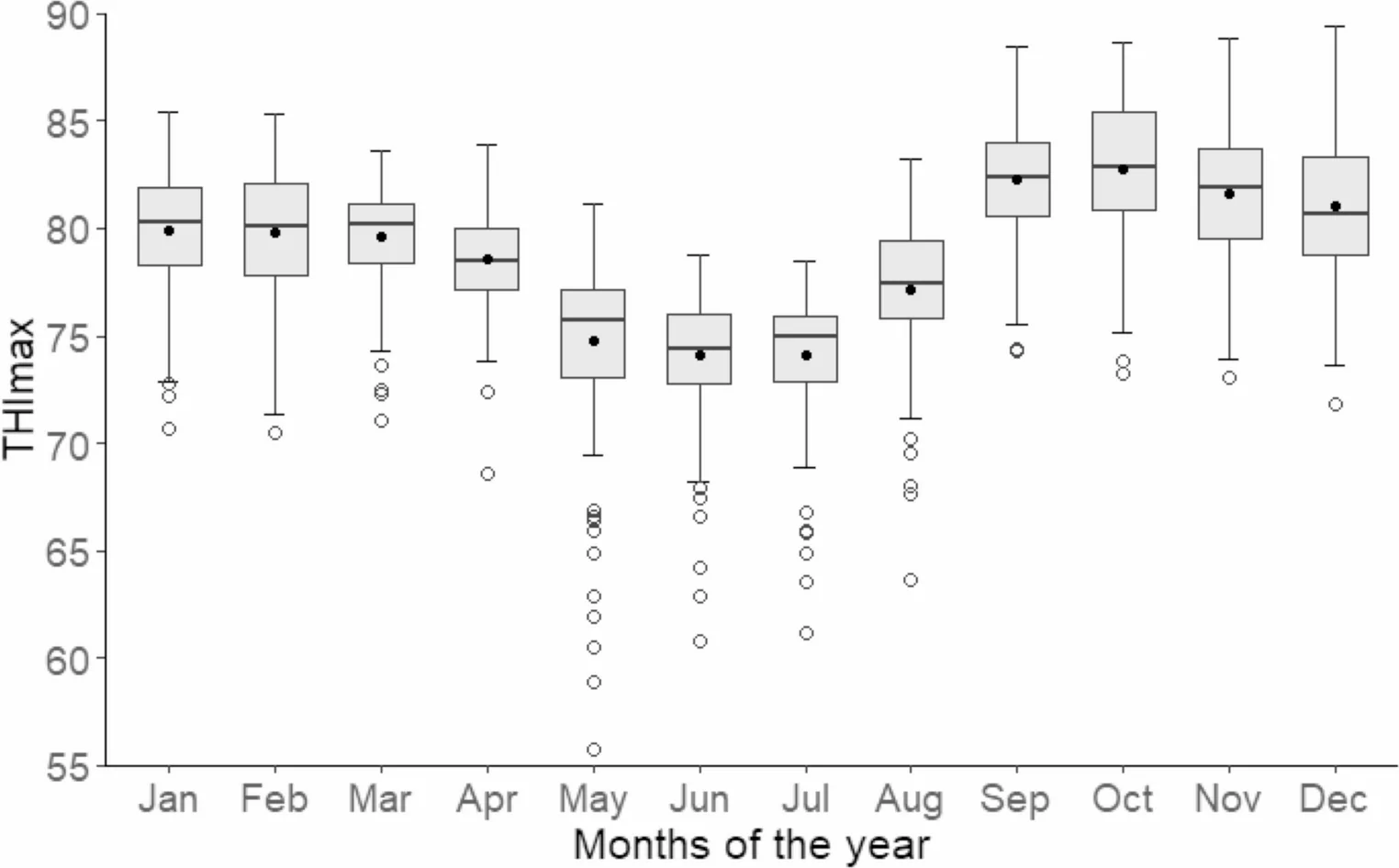
Box plot of the variation in the maximum Temperature-Humidity Index (THImax) by month from 2019 to 2023 in a tropical environment. The dot (●) represents the arithmetic mean

During the observation period, the mean THImax values were lowest in May, June, and July, with values of 74.8, 74.1, and 74.1, respectively. This indicates that most days were classified in the thermal comfort category (THImax ≤ 74) (Fig. 1). In the other months, the average THImax remained above 77, indicating moderate to severe heat stress conditions. From September to December, severe stress predominated, with THImax values averaging 82.2, 82.8, 81.6, and 81.0, respectively (Fig. 1).

**Fig. 2:**
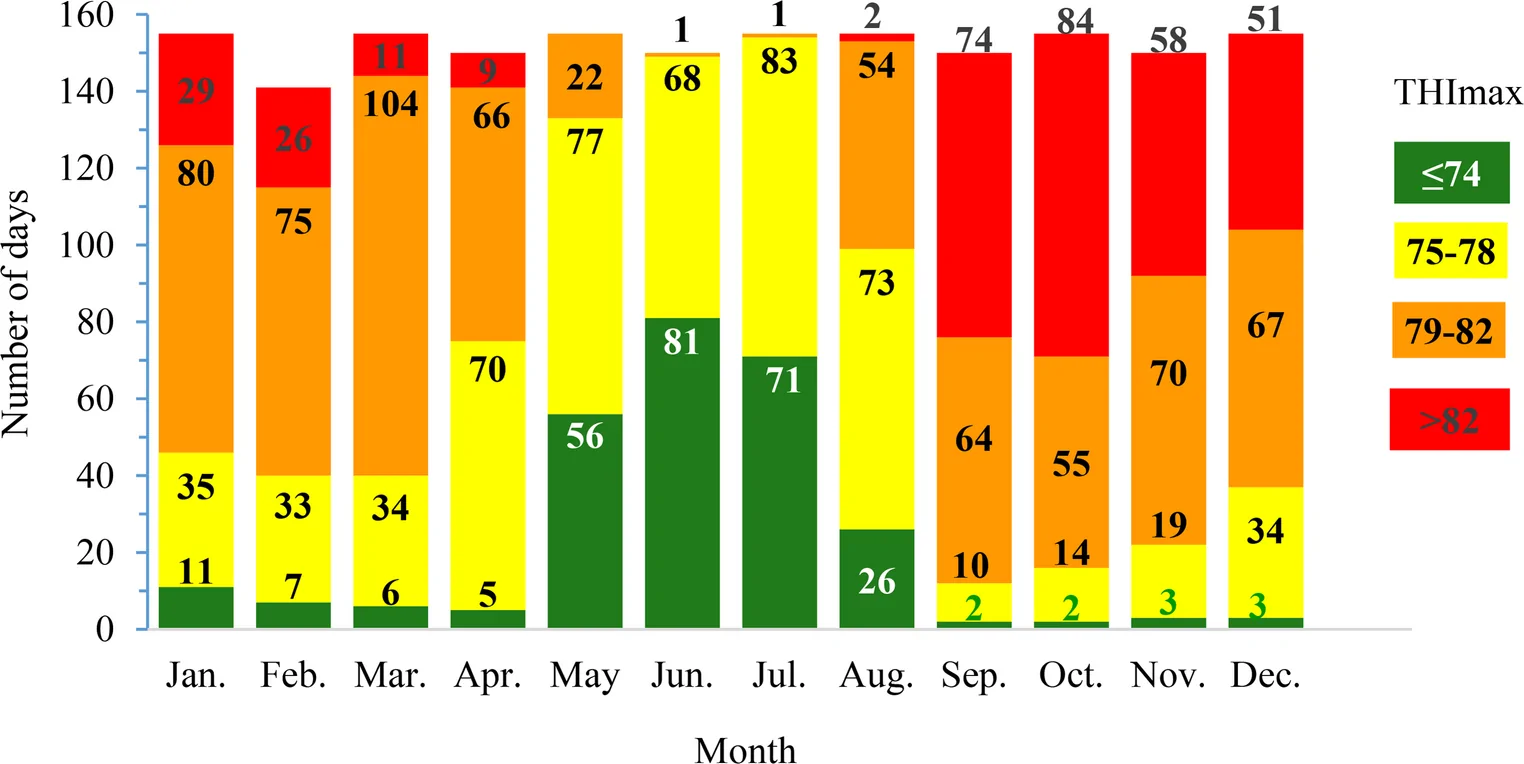
Monthly number of days within classes of maximum Temperature-Humidity Index (THImax) (≤74 (thermal comfort), 75–78 (mild stress), 79–82 (moderate stress), and >82 (severe stress)) from 2019 to 2023 in the Triângulo Mineiro region, Minas Gerais, Brazil

From January 2019 to December 2023, there were 273 days with thermal comfort (THImax ≤ 74), 550 days with mild stress (THImax 75–78), 659 days with moderate stress (THImax 79–82), and 344 days with severe stress (THImax > 82). May, June, and July had the highest number of days in thermal comfort (THImax ≤ 74), at 208 days. However, days classified as mild stress (THImax 75–78) occurred more frequently than days in thermal comfort, at 228 days (Fig. 2). In January, February, March, April, and August, days with mild and moderate stress predominated. In January, a total of 115 days were recorded; in February, 108 days; in March, 138 days; in April, 136 days; and in August, 127 days. From September to December, only 10 out of 610 days were classified as comfortable (THImax ≤ 74), while 267 days were classified as severe stress (THImax > 82), indicating a predominance of thermal stress conditions during this period (Fig. 2).

Quasi-Poisson regression analysis revealed that THImax had no significant impact on the number of stillbirths per day among gilts in any THImax category (*p* > 0.05) (Table 3). In primiparous sows, THImax had a significant negative effect in the comfort category (THImax ≤ 74) (*p* < 0.05), indicating that the lower the THImax, the higher the number of stillbirths per day. However, in the other THImax categories, the effect was not significant. An increase in the number of stillbirths was observed in females with 2–5 and ≥ 6 farrowings when births occurred during days of severe stress (THImax > 82) (*p* < 0.05) (Table 3). Therefore, sows with ≥ 2 farrowings are more sensitive to heat than gilts and primiparous sows.

**Table 3:** Estimated effect of maximum Temperature-Humidity Index (THImax) (≤ 74, 75–78, 79–82, and > 82) on the number of stillbirths per day on a commercial piglet farm in a tropical environment, estimated using a quasi-Poisson regression model

Parity group	Factors	≤ 74		75–78		79–82		> 82	
		Est. (± SE)	P-value	Est. (± SE)	P-value	Est. (± SE)	P-value	Est. (± SE)	P-value
Gilts	Int.	3.265 (1.55)	0.04	−3.452 (3.30)	0.30	−0.396 (0.02)	0.89	−0.449 (3.34)	0.89
	THImax	−0.025 (0.02)	0.23	0.063 (0.04)	0.14	0.022 (0.04)	0.53	0.023 (0.04)	0.55
Primiparous	Int.	4.957 (1.53)	< 0.01	−2.496 (3.60)	0.49	−3.178 (3.31)	0.34	0.743 (2.88)	0.80
	THImax	−0.055 (0.02)	0.01	0.045 (0.05)	0.33	0.053 (0.04)	0.19	0.005 (0.03)	0.88
2–5	Int.	4.419 (1.24)	< 0.01	3.311 (2.55)	0.20	4.123 (2.34)	0.08	−5.819 (2.25)	0.01
	THImax	−0.029 (0.02)	0.09	−0.012 (0.03)	0.70	−0.022 (0.03)	0.44	0.096 (0.03)	< 0.01
≥ 6	Int.	2.255 (1.65)	0.17	7.936 (3.33)	0.02	6.804 (2.88)	0.02	−3.767 (2.62)	0.15
	THImax	0.006 (0.02)	0.76	−0.080 (0.04)	0.07	−0.059 (0.04)	0.10	0.067 (0.03)	0.03

Int: intercept; SE: standard error; Est: estimate

**Fig. 3:**
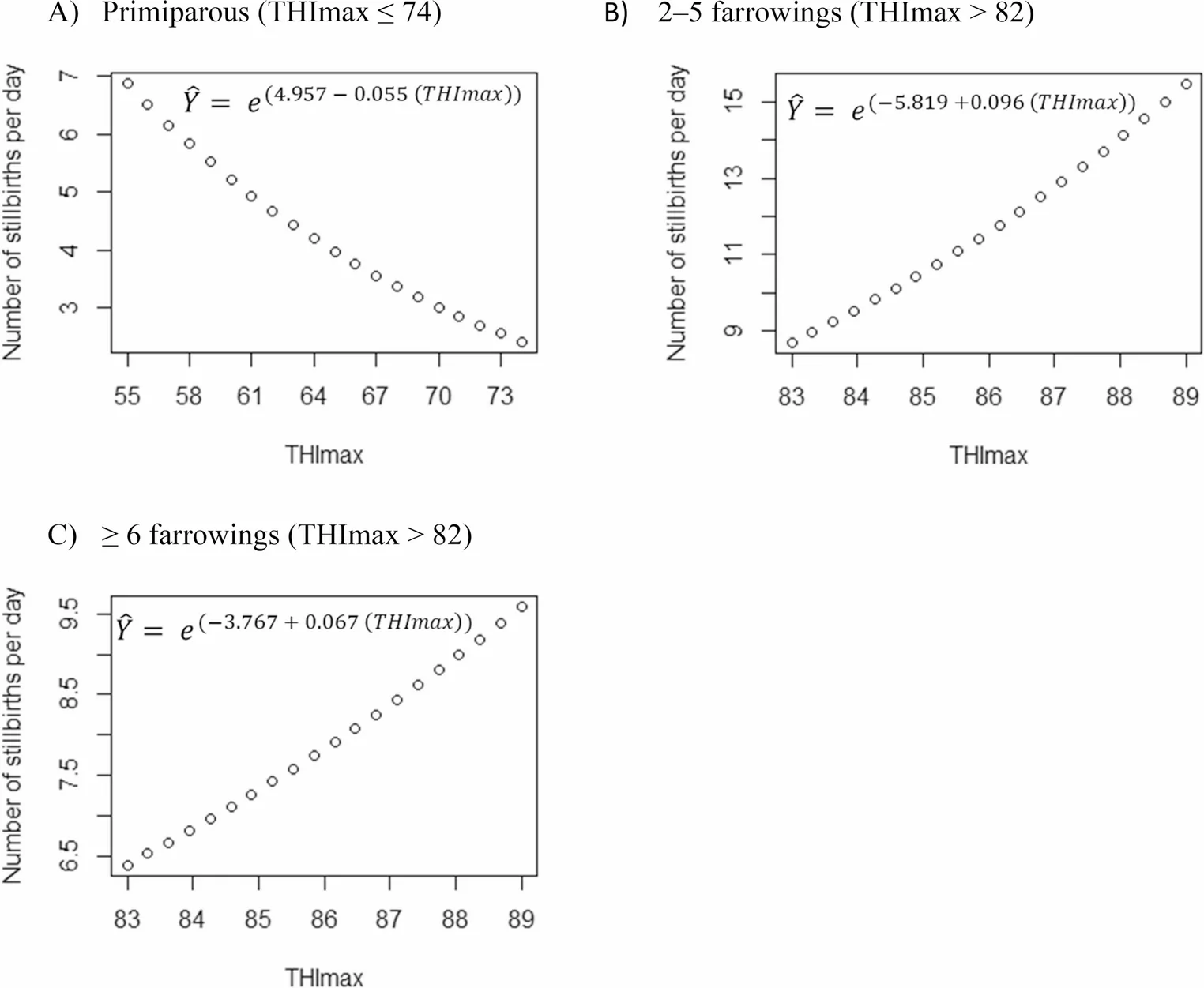
Relationship between THImax and number of stillbirths per day in significant quasi-Poisson models for different parity groups: (A) primiparous (THImax ≤ 74), (B) 2–5 farrowings (THImax > 82), and (C) ≥ 6 farrowings (THImax > 82)

Using the equations to estimate the average number of stillbirths per day revealed that primiparous sows that farrowed on days with a THImax of 55 had a predicted average of 7 stillbirths per day. However, on days with a THImax close to 74, the average daily number of stillbirths decreased to approximately 2–3 (Fig. 3 A). Thus, within the comfort classification (THI ≤ 74), values closer to 74 were associated with a lower mean number of stillbirths per day. It is noteworthy that days with THI values close to 55 are rare in the study region.

For sows with 2–5 farrowings, an increase in THImax from 83 to 89 resulted in a significant increase in the daily average number of stillbirths, from approximately 9 to 15 (Fig. 3B). This increase suggests that severe heat stress significantly impacts the number of stillbirths in this parity group. The same trend is observed for sows with ≥ 6 farrowings, but with less intensity. On days with a THImax of 83, the average number of stillbirths per day was approximately 7. On days with a THImax of 89, this number increased to 10 (Fig. 3C). While the impact of extreme heat is significant in this group as well, the increase in stillbirths per day is less pronounced than in sows with 2–5 farrowings.

**Fig. 4:**
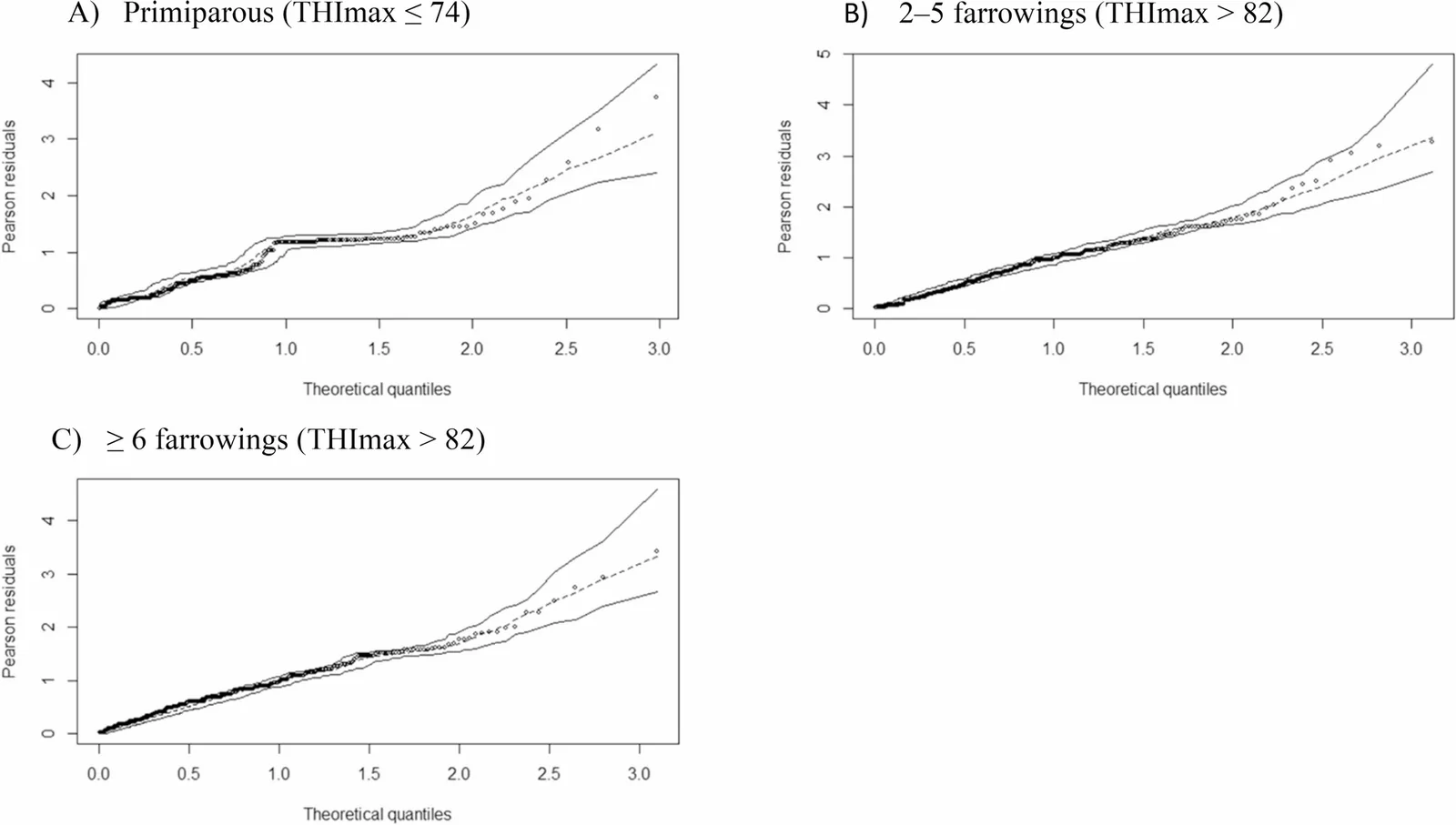
Half-normal plot of significant quasi-Poisson models for (A) primiparous sows with THImax ≤ 74, (B) sows with 2–5 farrowings with THImax > 82, and (C) sows with ≥ 6 farrowings with THImax > 82

Pearson’s residual statistics show that the quasi-Poisson models indicating an effect of THImax on the number of stillbirths per day were adequately fitted. This was assessed using half-normal graphs (Fig. 4), which compare observed residuals with expected values. Most points remained within the simulated envelope, indicating that the residuals follow the expected pattern for an adequate model at a 95% confidence level.

When analyzing the average number of stillbirths per farrowing, the number remained stable in gilts and females with 2–5 farrowings among the THImax categories of ≤ 74, 75–78, and 79–82, but increased significantly in the > 82 category, indicating a more pronounced effect of severe stress (Table 4). The lowest average number of stillbirths per farrowing in primiparous sows was observed in the THImax 75–78 category, while the highest number was observed in the THImax > 82 category (Table 4). In sows with ≥ 6 farrowings, the lowest mean number of stillbirths was observed under thermal comfort conditions (THImax ≤ 74), whereas the highest mean value occurred at THImax ≥ 79 (Table 4).

**Table 4:** Mean number of stillborn piglets per farrowing under different THImax categories (≤ 74, comfort – C; 75–78, mild stress – MS; 79–82, moderate stress – MoS; >82, severe stress – SS) and the Stress/Comfort ratio (S/C)

Parity group	Mean stillbirths*				S/C ratio**		
	≤ 74 (C)	75–78 (MS)	79–82 (MoS)	> 82 (SS)	MS/C	MoS/C	SS/C
Gilts	0.96a	0.96a	0.99a	1.15b	1.00	1.03	1.20
Primiparous	0.81ab	0.74a	0.81ab	0.87b	0.91	1.00	1.07
2–5	1.03a	1.04a	1.07a	1.17b	1.01	1.04	1.14
≥ 6	1.28a	1.39ab	1.44b	1.52b	1.09	1.13	1.19

*Means followed by different letters in the rows (by parity group) differ according to Games Howell test (*p* < 0.05)

**The S/C ratio of 1.0 does not differ between the stress (S) and comfort (C) periods

THI the greatest impact of heat stress, with an increase in the mean number of stillbirths per farrowing from 1.28 (THImax ≤ 74) to 1.52 (THImax > 82) (Table 4).

In all parity groups, the SS/C ratio was greater than 1.0, indicating an increase in stillbirths under severe stress conditions (THImax > 82) (see Table 4). Under severe stress conditions, the average number of stillbirths increased by 20%, 14%, and 19% in gilts with 2–5 and ≥ 6 farrowings, respectively, compared to comfortable conditions (≤ 74). Conversely, in primiparous sows, the effect was less pronounced, with an increase of only 7%, indicating lower sensitivity to heat stress compared to other parity groups. Additionally, the MS/C ratio was less than 1.0, indicating a 9% reduction in stillbirths during mild stress days (Table 4).

## Discussion

The present study was conducted in a tropical environment where THImax was frequently above the thermal comfort zone for sows (THImax > 74). The percentage of stillbirths per farrowing ranged from 5.73% to 10.41%. Higher values were observed in sows with ≥ 6 farrowings, while lower values were observed in primiparous sows. A study conducted in Thailand reported an average stillbirth rate of around 7.3%, ranging from 5.1% to 8.2% among herds (Udomchanya et al. 2019). This rate has increased due to genetic selection for higher prolificacy. Genetic selection for high prolificacy affects the reproductive physiology and welfare of sows and piglets, promoting changes in birth weight and farrow size. These changes are associated with a higher incidence of stillbirths in pigs (Reproto 2020). An increase in the number of live piglets requires adaptations in uterine capacity, as well as in placental and fetal functions. Intrauterine crowding and competition for space and nutrients compromise piglet vitality and farrow uniformity (Kemp et al. 2018; Reproto 2020). Recent results confirm this pattern: the average number of piglets born per farrow in hyperprolific herds was 14.1, with a stillbirth rate of 6.7%. This rate was higher in females with ≥ 17 total piglets per farrow (10.9%) than in those with ≤ 13 (6.1%) (Ngo et al. 2024). The findings of the present study, in which the average number of piglets born per farrow was also 14.1, align with values reported for hyperprolific herds in tropical regions. These findings suggest that factors such as the age of the dam and heat stress may contribute to an increase in stillbirths, in addition to farrow size.

The reproductive potential of sows varies by age. Gilts and primiparous sows tend to have moderate reproductive performance. Sows with 2–5 farrowings demonstrate their maximum potential. Older sows (parity ≥ 6) have reduced productivity (Adi et al. 2022, 2024). Gilts and primiparous sows have a lower number of piglets born, lower total litter weight, and greater litter uniformity compared with multiparous sows (Buthelezi et al. 2024). Increased farrow size, especially in multiparous sows (≥ 2 farrowings), is associated with lower farrow uniformity at birth and a higher incidence of stillbirths (Buthelezi et al. 2024). Additionally, hyperprolific sows with ≥ 7 farrowings tend to have longer farrowing periods and require more assistance, which contributes to a higher incidence of stillbirths (Adi et al. 2024; Wongwaipisitkul et al. 2024). The stillbirth rate positively correlates with farrowing assistance and negatively correlates with average birth weight (Feyera et al. 2018). In the present study, the occurrence of stillbirths was found to differ according to parity group, being lower in younger dams and higher in older ones. The effect of heat on the number of stillbirths also differed between parity groups. These differences may be due to physiological development, reproductive maturity, and thermoregulatory capacity. Gilt and primiparous sows have immature endocrine and immune systems and high energy demands for growth and development, which may make them more susceptible to lower temperatures at the end of gestation (Tani et al. 2016; Buthelezi et al. 2024).

The meteorological data yielded results that were consistent with previous findings regarding the annual variation of THI in tropical regions. These results evidenced the predominance of values that were above the thermal comfort zone for sows (THImax > 74) throughout most of the year (Machado et al. 2016; Brito et al. 2022). Of the 1,826 days evaluated, only 273 (14.95%) had thermal comfort conditions (THImax ≤ 74). Of the 606 days in the summer months (December to March), only 27 days had THImax ≤ 74. In contrast, in a temperate environment, THImax follows a well-defined seasonal pattern, with high values concentrated in the summer (Wegner et al. 2014). When analyzing three years of data from May to September in Germany, Wegner et al. (2016) found that 403 of 459 days (88.45%) were thermally comfortable (THImax ≤ 74). This means that the breeding stock remained in thermal comfort 88.45% of the time, even in summer. These data demonstrate the contrast with tropical regions, where exposure to heat stress is constant throughout the year and poses a persistent challenge to the reproductive management of breeding pigs. In such regions, adopting technological solutions is a crucial strategy for mitigating the effects of heat stress. However, most pig farms in Brazil do not have air conditioning systems, indicating limitations in reducing the impact of high temperatures on pig production.

Sows exposed to elevated ambient temperatures respond differently according to parity. Gilts and primiparous sows produce less heat compared with multiparous sows, as the latter exhibit higher feed intake, body weight, and metabolic rate (Cabezón et al. 2017). In addition, thermal discomfort can increase the number of stillbirths in multiparous sows by elevating cortisol concentrations. Elevated cortisol inhibits the release of oxytocin and reduces the effectiveness of uterine contractions, prolonging the duration of farrowing (Edwards 2002; Vanderhaeghe et al. 2010; He et al. 2019). Multiparous sows are more susceptible to uterine dysfunction, prolonged farrowing, and complications associated with increased expulsion effort, all of which contribute to an increased number of stillbirths, particularly under heat stress conditions (Vanderhaeghe et al. 2010; Ngo et al. 2024). Quasi-Poisson regression model results confirmed this pattern, indicating that THImax significantly impacts the increase in stillbirths in multiparous sows farrowing on days of severe stress (THImax > 82). The model predicted an increase in the average number of stillbirths per day from 9 to 15 in sows with 2 to 5 farrowings and from approximately 7 to 10 in sows with ≥ 6 farrowings as THImax increased from 83 to 89. This evidence shows reduced productivity and consequent economic impacts due to heat stress.

However, young females exhibited different patterns in response to THImax with regard to the number of stillbirths per day. According to the quasi-Poisson regression model, no significant association was observed between THI and the number of stillbirths per day in gilts. In a study conducted in a temperate environment, however, Zhao et al. (2022) reported that gilts exposed to heat stress before farrowing exhibited reduced oxygen supply to piglets via the placenta due to umbilical cord rupture. This resulted in a higher stillbirth rate. The results of the present study may indicate variation in individual responses in sows since the model considered the number of stillbirths per day. According to the quasi-Poisson model, the predicted values obtained from the model equation indicated that, within the THI range of 55 to 74, primiparous sows showed a reduction in the number of stillbirths per day as THI increased toward 74. Tani et al. (2016) reported in a study in a temperate environment that the effects of low temperature (0–5 °C) on stillbirths were greater in gilts and primiparous sows. The authors suggest that exposing young sows to low temperatures may prolong the duration of farrowing and increase the risk of stillbirths, similar to what occurs in heat-stressed multiparous sows. Additionally, they emphasize that prolonged or intermittent asphyxia, both intrauterine and during farrowing, compromises the vitality of piglets and their ability to respond to cold stress.

Second-litter syndrome is characterized by reduced reproductive performance in the second litter compared to the first (Nam et al. 2024). Reduced reproductive performance in primiparous sows may be related to nutritional deficiencies during the first lactation. Insufficient energy intake during this period may not meet the female’s high demands, leading to the mobilization of body reserves and weight loss (Schenkel et al. 2010). The mobilization of dorsal fat reserves during lactation has been associated with an increased stillbirth rate, reduced litter size, and impaired development in subsequent births (Tummaruk et al. 2022). These factors may explain why primiparous females are more vulnerable to THImax conditions < 74. The loss of body reserves can compromise the thermoregulatory capacity of homeothermic animals because subcutaneous tissue acts as a thermal insulator (Gómez-Prado et al. 2022). In the present study, estimated by regression model quasi- Poison primiparous sows that farrowed on days with a THImax of 55 had an average of approximately 7 stillbirths per day, while on days with a THImax close to 74, the average number of stillbirths per day decreased to approximately 3. This result indicated that close to THImax of 74 there is a decrease in the number of stillbirths in primiparous sows.

The present study shows that sows that farrowed under severe stress (THImax > 82) had a higher average number of stillbirths per farrowing, regardless of the parity group. Several authors have reported that heat stress compromises uterine contractility, increases the risk of fetal hypoxia, and reduces neonatal viability, thereby increasing the incidence of stillbirths (Edwards 2002; He et al. 2019). Considering the average number of stillbirths per farrow, the ratio of severe stress to thermal comfort (SS/TC) was found to increase stillbirths by 20%, 7%, 14%, and 19% for gilts, primiparous sows, sows with 2–5 farrowings, and sows with ≥ 6 farrowing, respectively. These results demonstrate that the average number of stillbirths per farrowing significantly increases when females are exposed to intense heat stress, particularly in gilts and multiparous sows. These results reinforce the findings of quasi-Poisson modeling, which indicated greater vulnerability to heat stress in females in a higher parity group. The results also suggest that gilts may be affected differently. Based on the results of this study, the S/C ratio is a useful tool for quantifying the effects of heat stress on the reproductive performance of sows in tropical environments. Applying the S/C ratio allows one to identify production losses associated with heat stress, demonstrating its potential as an applicable indicator for sows of different parity groups under tropical conditions. Therefore, the S/C ratio can guide pig farmers in managing the thermal environment of sows in intertropical regions to mitigate the harmful effects of heat.

It is important to emphasize that the present study is observational, as it was based on data obtained from a commercial farm and meteorological data retrieved from the NASA/POWER database. In addition, nutrition, management, genetics, and other unmeasured factors may have influenced the results. However, despite these limitations, the regression models used were appropriate for the database according to the model fit results using Pearson’s residuals, which ensured their reliability. Thus, the generated equations can be used to estimate the effect of THImax on the number of stillbirths per day in multiparous (≥ 2 farrowings) sows under THImax > 82 and in primiparous sows under THImax ≤ 74.

The S/C ratio is also a useful tool for pig farmers because it is easy to apply and allows the impact of heat stress on stillbirths to be quantified compared to thermal comfort conditions. Considering different categories of THImax and parity group, the S/C ratio can identify more vulnerable groups and periods of higher risk, contributing to more efficient management strategies. However, is important to note that is a descriptive indicator, therefore, it should be used together with other variables to monitor the thermal environment and productive performance.

The findings of this study therefore highlight the importance of considering parity group when developing heat stress mitigation strategies, particularly in tropical regions where fluctuations in THImax throughout the year can compromise reproductive efficiency and the economic viability of production systems. Further studies are warranted to evaluate the effects of THI using air temperature and humidity measured inside the farrowing facility at the time of parturition.

## Conclusions

Our study indicates that THImax appears to influence the occurrence of stillbirths in sows, with effects varying according to parity. Quasi-Poisson regression analysis revealed that a THImax > 82 on the day of farrowing is associated with an increase in stillbirths among multiparous females (≥ 2 farrowings). In primiparous sows, the regression model suggests that THImax values < 74 on the day of farrowing are associated with a higher number of stillbirths per day, with a reduction as THImax approaches 74. The severe stress/comfort ratio was greater than 1 for all parity groups, suggesting an increase in the mean number of stillbirths per farrowing. The effects were more pronounced in gilts and multiparous females.

## Data Availability

The datasets generated and/or analyzed during the current study are not publicly available.
